# DeepDoublet identifies neighboring cell-dependent gene expression

**DOI:** 10.1186/s44342-024-00031-2

**Published:** 2024-12-18

**Authors:** Linbu Liao, Junyoung Kim, Kanghee Cho, Junil Kim, Byung-Kwan Lim, Kyoung Jae Won

**Affiliations:** 1 Cancer Institution, Cedars-Sinai Medical Center, Los Angeles, CA USA; 2 Department of Bioinformatics, Soongsil University, Seoul, Korea; 3 School of Systems Biomedical Science, Soongsil University, Seoul, Korea; 4 Department of Biomedical Science, Jungwon University, Goesan-Gun, Chungbuk, Korea; 5 Department of Computational Biomedicine, Cedars-Sinai Medical Center, Los Angeles, CA USA

**Keywords:** Doublet decomposition, Cell–cell interaction, Deep learning, Single-cell RNA sequencing

## Abstract

Cells interact with each other for proper function and homeostasis. Often, co-expression of ligand-receptor pairs from the single-cell RNAseq (scRNAseq) has been used to identify interacting cell types. Recently, RNA sequencing of physically interacting multi-cells has been used to identify interacting cell types without relying on co-expression of ligand-receptor pairs. This opens a new avenue to study the expression of interacting cell types. We present DeepDoublet, a deep-learning-based tool to decompose the transcriptome of physically interacting two cells (or doublet) into two sets of transcriptome. Applying DeepDoublet to the doublets of hepatocyte and liver endothelial cells (LECs), we successfully decomposed into the transcriptome of each cell type. Especially, DeepDoublet identified specific expression of hepatocytes when they are interacting with LECs. Among them was Angptl3 which has a role in blood vessel formation. DeepDoublet is a tool to identify neighboring cell-dependent gene expression.

**Supplementary Information:**

The online version contains supplementary material available at 10.1186/s44342-024-00031-2.

## Introduction

Cells interact with other cells continuously to maintain homeostasis and function properly [1, 2]. During development, for instance, cell communication plays a role in specifying fates including gonadogenesis, vulval, and neurogenesis [3]. This suggests that a cell and its transcriptome can be influenced by the surrounding microenvironment.

Single-cell RNA sequencing (scRNAseq), by revealing transcriptomic information of a cell, provides information about cell heterogeneity [4]. Various clustering algorithms [5, 6] were applied on scRNAseq data to identify cell types and the associated marker genes. However, it is still not enough to study the transcriptomic changes due to the surrounding microenvironment.

We hypothesize that cell interaction can change the transcriptome of a cell. A well-known example is the co-expression of ligands and receptors of the interacting cell types [7]. However, there are diverse ways of cell communications besides ligand-receptor including direct cell communication through gap junctions [8, 9]. It is still difficult to understand the gene expression changes due to cell interaction as the neighboring cell information is lost in the scRNAseq.

Recently, the transcriptome of physically interacting multi-cells was measured to identify interacting cell types without relying on ligand-receptor expression pairs. ProximID identified the interacting cell types in the bone marrow and small intestine in mice after mildly dissociating cells to obtain two or more cell clumps [10]. ProximID identified the interaction of Lgr5-expressing stem cells with Tac1-expressing enteroendocrine cells in murine intestine. ProximID employed a random forest approach to predict interacting cell types [10]. Physically interacting cells followed by sequencing (PIC-seq) was applied to interrogate interactions of immune and epithelial cells in neonatal murine lungs [11]. T cells engaged in interaction with epithelial cells showed gene expression statistically different from the cells not engaged in the interactions. Paired-cell RNA sequencing (pcRNAseq) [12] exclusively selected the interacting cells of liver endothelial cells (LECs) and hepatocytes by sorting cells based on size and CD31, a marker for endothelial cells [13]. pcRNAseq has been applied to identify the zonational expression of LECs with the guide of the zonational expression markers in hepatocytes.

The physically interacting multi-cells have mainly been used to identify interacting cell types. However, the expression of individual cells comprising the interacting cells was not well studied. To determine the transcriptome of two-interacting cells, we developed DeepDoublet, a deep-learning-based method that determines two single cells comprising a doublet. DeepDoublet was trained with artificial doublets and was tested in the public pcRNAseq data. With the prediction of DeepDoublet, we further identified genes that are differentially expressed in hepatocytes when interacting with endothelial cells.

## Materials and methods

### Data description and preprocessing

Single-cell [12, 14] and pcRNAseq [12] data in adult mouse livers were used for this work. The data includes 1415 hepatocytes, 1203 LECs, and 4602 doublets (hepatocyte-LEC). From the 1415 hepatocytes and the 1203 LECs, 400,000 artificial doublets were generated to train the models. Genes with at least 1 UMI in at least 4% of cells of each cell type were selected. In total, 8676 genes were used for model training.

### Artificial doublet

Artificial doublets were obtained by randomly mixing UMI count vectors of hepatocytes and LECs:

$${ArtUMI}_{AB}= \alpha \times {UMI}_{A}+(1-\alpha) \times { UMI}_{B}$$

$$\alpha \epsilon [0.1, 0.2, 0.3, 0.4, 0.5, 0.6, 0.7, 0.8, 0.9]$$

where *ArtUMI*_AB_, *UMI*_A_, and *UMI*_B_ denote gene expression vectors of an artificial AB doublet, a cell with type A and a cell with type B respectively. $\alpha$ is a mixing factor randomly selected from the nine values.

### Deep-learning-based decomposition

Two three-layer FCNNs were used to decompose hepatocyte-LEC doublet. The numbers of nodes for the three layers are 2048, 1024, and 512 in sequence. Doublets were normalized to counts per million mapped reads/fragments (CPM) before feeding them into neural networks. Categorical cross entropy [15] was used as the loss function. The Adam algorithm [16] was used to optimize the two FCNNs. The final model was trained on all artificial doublets. The value of the *j*-th node in the *i*-th layer of an FCNN was calculated as follows:

$${x}_{ij}=\sigma ({w}_{ij}{x}_{i-1})$$

where ${w}_{ij}$ is the *j*-th row of the *i*-th weight matrix, ${x}_{i-1}$ is the node vector of the (*i*−1)-th layer, and $\sigma$ represents activation function. The rectified linear unit (ReLU) function is the activation function for the first three layers, while the softmax function is the function for the last layer.

$$ReLU\left(z\right)=\text{max}\left(0,z\right)$$

$$softmax(z_i)=\frac{e^{z_i}}{\sum_{j=1}^Ke^{z_j}}\text{for i}=1,\dots,K,$$

where *K* is the number of nodes in the output layer.

### Logistic regression

Logistic regression was implemented using scikit-learn [17] package in Python with default parameters for multiclass classification.

### Differential expression analysis

By applying the 2 trained neural networks to 4602 real hepatocyte-LEC doublets, 2 score vectors corresponding to all hepatocytes and all LECs were assigned to each doublet. The sum of each vector equals to 1 as the softmax function was used as the activation function. We selected hepatocytes and LECs that were scored more than 0.01 for downstream analysis. Most of the selected cells were expected to be involved in hepatocyte-LEC interaction. Multiple cells will be selected in LECs and hepatocytes, respectively. Subsequently, Wilcoxon-Mann–Whitney test [18] was applied between the selected hepatocytes and the other unselected hepatocytes on all the available genes. We assumed that the selected hepatocytes interact with LECs and selected LEC interact with hepatocytes.

When selecting significant genes, the adjusted *p*-value [19] 0.05 was set as the cutoff for Wilcoxon-Mann–Whitney test. Logarithmic fold change was calculated in this way:

$$Log\left(FC\right)={log}_{2}^{\frac{Mean\left({Exp}_{k1}\right) + b}{Mean\left({Exp}_{k2}\right) + b}}$$

where “$Mean\left({Exp}_{k1}\right)$” is the mean expression value of gene *k* for the cell group 1 and “$Mean\left({Exp}_{k2}\right)$” is the mean expression value of gene *k* for the cell group 2. *b* is a constant factor to alleviate the importance of lowly expressed genes. It is set to 1 in our analysis. The gene expression data used was processed with the Scanpy [19] preprocessing pipeline. The gene expression values were normalized to 10,000. Then, the normalized values were transformed with natural logarithmic function:

$${Exp}_{log}={log}_{e}^{{Exp}_{norm}+1}$$

Here, a constant value 1 was added to each normalized expression value to avoid the case that the expression value is 0. After logarithmic transformation, we regressed out the effects of total gene counts for each cell and scaled gene expression across all cells using Scanpy preprocessing functions [19].

### Software used for analysis and model training

Single-cell and pcRNAseq data analysis was performed with Scanpy 1.6.0. Clustering was done using Leiden algorithm [20] implemented in Scanpy 1.6.0. Deep learning was implemented using Keras 2.2.4 [15] with TensorFlow-GPU 1.15.0 [21] as the backend in Python 3.7.4. Logistic regression was implemented using scikit-learn 0.24.1 in Python 3.7.4.

### Tissue staining

Mouse liver was snap-frozen by embedding in O.C.T. Tissue-Tek (Sakura Finetechnical, Torrance, CA, USA) after 25% sucrose treatment using isobutane chilled in dry ice. A 7-μm sections were cut by Leica cryostat (Leica, Bannockburn, IL, USA). Specimens were fixed with ice-cold acetone, followed by blocking and permeabilization with 2% BSA and 1% Triton X-100 in PBS, and incubated with primary antibodies against Angptl3 (1:100; Santa Cruz, CA, USA) and PECAM (1:500; R&D System, MN, USA). The target proteins were visualized with the secondary antibodies conjugated with fluorescence (Alexa Fluor 488 and 594, 1:400; Invitrogen, CA, USA) and Hoechst nuclear stain. Fluorescence images were taken and processed using a fluorescent microscope (Olympus, CA, USA) [22].

### Performance evaluation

We defined true positive (TP) when the model correctly predicts a positive case. A false positive (FP) happens when the model incorrectly identifies a negative case as positive. A true negative (TN) arises when the model correctly predicts a negative case. Finally, a false negative (FN) occurs when the model incorrectly identifies a positive case as negative. Then,

$$\begin{array}{c}\mathrm{Accuracy}\;=\;(TP+TN)\;/\;(TP+TN+FP+FN),\\FPR\;=\;FP\;/\;(FP+FN),\;\mathrm{and}\\FNR\;=\;FN\;/\;(TP+FN).\end{array}$$

## Results

### Architecture of DeepDoublet

**Fig. 1:**
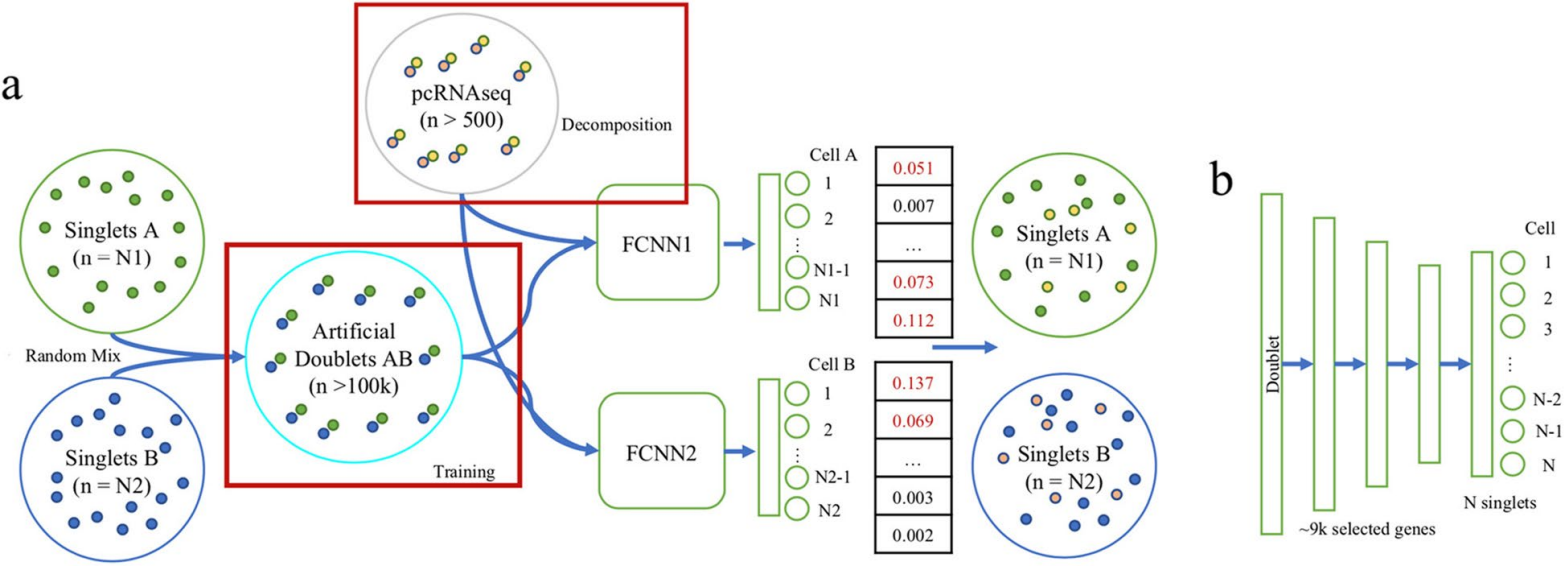
The architecture of DeepDoublet. **a** Main workflow of DeepDoublet. The mixture of two cells (A type and B type) was used to train two FCNNs prepared for each cell type. The output is the index of the cell used to form the mixture. Once trained, each FCNN selects the output nodes that have a value higher than a cutoff. **b** The structure of an FCNN

DeepDoublet is a tool for identifying single cells that comprise a pair of interacting cells. DeepDoublet is composed of two fully connected neural networks (FCNNs) with three hidden layers besides input and output layers (Fig. 1). Each FCNN is assigned to a cell type. Two FCNNs receive the whole transcriptome of the mixture of cells and predict the single cells that best match the mixed transcriptome. Therefore, the number of output nodes for FCNN_1_ and FCNN_2_ is the number of single cells used for each cell type.

DeepDoublet is trained using artificial doublets by mixing transcriptome from single cells with diverse proportions. Once trained, DeepDoublet determines the cells for the mixture of transcriptome or RNAseq from multi-cells.

### Validation using artificial datasets

As pcRNAseq has the information of two interacting cells, we used pcRNAseq [12] and the scRNAseq for hepatocytes [14] and LECs [12] to evaluate the performance of DeepDoublet. First, we performed fivefold cross-validation on the 400,000 artificial doublets that were generated by randomly mixing 1415 hepatocytes and 1203 LECs with various proportions. In this scheme, the artificial doublets used during training are not used for testing. We compared the performance of DeepDoublet with the logistic regression (LR) and Naïve Bayes [23]. For the test, we calculated the number of correct predictions of cells over the total number of predictions.

Both DeepDoublet and the LR showed almost perfect prediction in predicting hepatocytes (accuracy > 0.995) (Table 1), while the prediction performance for LECs was undermined, especially for the LR. Naïve Bayes performed worse in our simulation. DeepDoublet also showed best scores when evaluated with additional FPR and FNR. To further understand the advantages of DeepDoublet, we interrogated the distribution of unique molecular identifier (UMI) for LECs and hepatocytes (supplementary Fig. 1). LECs had a significantly low number of UMIs compared with hepatocytes. The LR may have difficulties in identifying the interacting cells due to the low information contents in LECs.

**Table 1:** Performance comparison between DeepDoublet and the LR. Fivefold cross-validation was conducted on 400,000 artificial doublets

	DeepDoublet		LR		Naïve Bayes	
Cell type	Hepatocyte	LEC	Hepatocyte	LEC	Hepatocyte	LEC
Accuracy	0.998	0.842	0.996	0.687	0.871	0.789
FPR	1.82 × 10^−6^	1.08 × 10^−4^	4.10 × 10^−3^	0.262	1.00 × 10^−3^	2.00 × 10^−4^
FNR	2.59 × 10^−3^	0.130	4.20 × 10^−3^	0.314	0.13	0.211

### DeepDoublet identifies neighboring cell-dependent gene expression

**Fig. 2:**
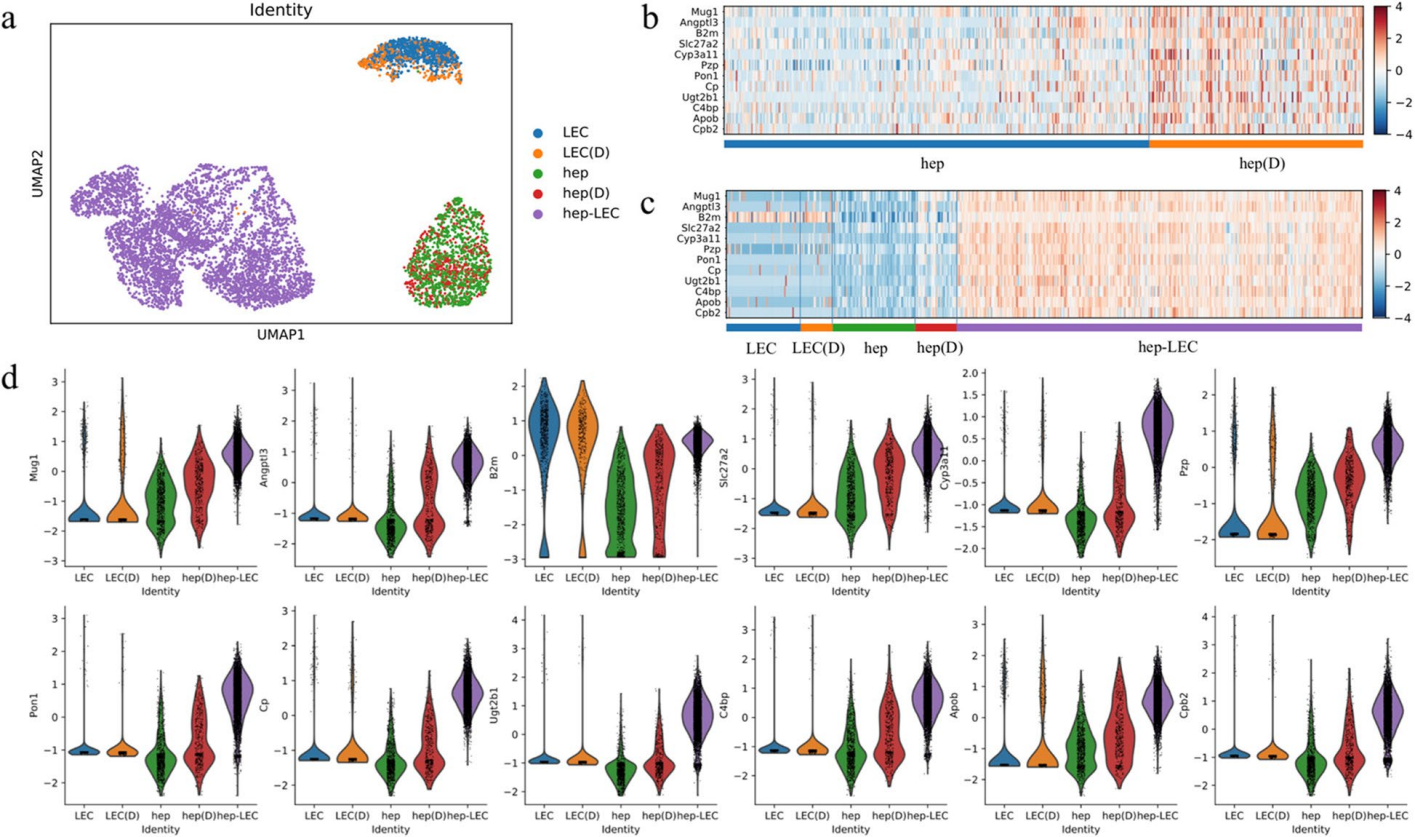
DeepDoublet predicts neighboring cell-dependent gene expression.** a** A UMAP plot of hepatocytes, LECs, and hepatocyte-LEC doublets. Hepatocytes interacting with LECs are labeled as hep(D), and LECs interacting with hepatocytes are with LEC(D). **b** The top 12 genes upregulated in the hepatocytes interacting with LECs. **c** The top 12 genes across various cell types or in pcRNAseq (hep-LEC). **d** The violin plots for the top 12 genes

We applied DeepDoublet to the pcRNAseq (4602 clumps) data comprised of 1 hepatocyte and 1 LEC. We used scRNAseq of the hepatocytes [14] and LECs [12] and predicted which cells among them comprise pcRNAseq. We labeled hepatocytes interacting with LECs as hep(D) and LECs interacting with hepatocytes as LEC(D). The Uniform Manifold Approximation and Projection (UMAP) [24] for hepatocytes, LECs, and the pcRNAseq showed that hepatocytes interacting LECs were not distinguishable using the naive clustering approach (Fig. 2a).

We investigated the genes specifically expressed in cells that interact with LECs (Fig. 2b). We found 25 genes including Angtl3 and Mug1 that were highly expressed in the hepatocytes interacting with LECs and 32 downregulated genes compared with other hepatocytes (supplementary Tables 1 and 2, adjusted *p*-value < 0.05 and |logFC|> 0.5). However, we did not find differentially expressed genes for LECs when interacting with hepatocytes. The heatmap and the violin plots for the top 12 genes show that genes such as Angptl3, Cyp3a11, Pon1, and Cp were highly expressed in the hepatocytes interacting with LECs and in the cell clumps from pcRNAseq compared with hepatocytes or LECs (Fig. 2c, d). Among them, Angptl3 is known to have a role in blood vessel formation [25, 26], suggesting its role next to endothelial cells. The expression of Angptl3 is low both in hepatocytes and LECs but high in hepatocytes interacting with LECs (hep(D)) and the pcRNAseq (hep-LEC).

### Hepatocytes proximal to LECs show higher expressions in Angptl3

**Fig. 3:**
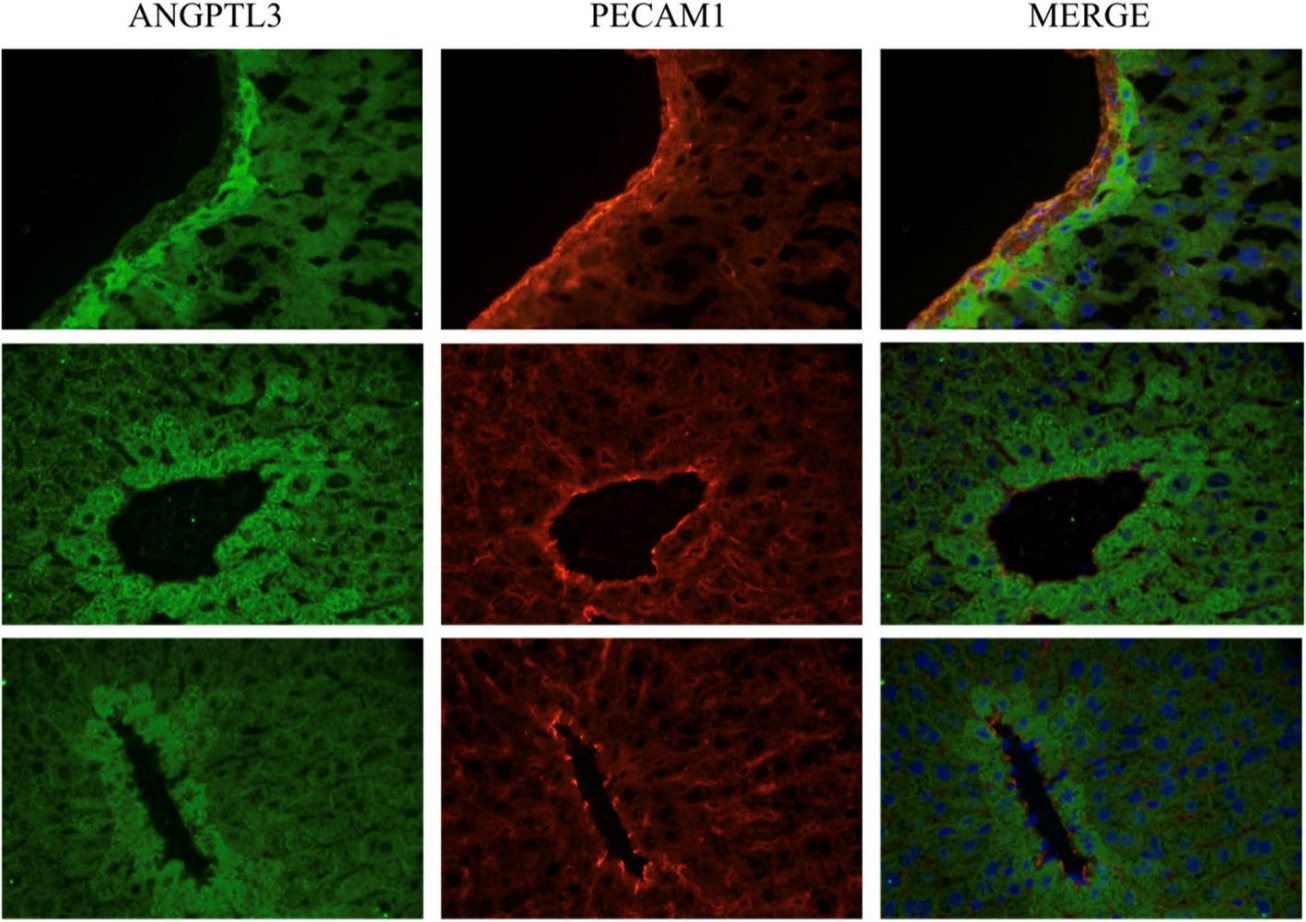
ANGPTL3 is expressed in the hepatocytes contacting with LECs. ANGPTL3 is more highly expressed in the hepatocytes close to LECs

To validate our observation, we performed staining analysis using Angptl3 and PECAM, an endothelial cell marker (Fig. 3). We selected three regions in an adult mouse liver where blood vessels are shown. We found stronger signals for Angptl3 at the regions where blood vessels are located (marked by PECAM). Hepatocytes distant to blood vessels showed weaker signals of Angptl3, validating that Angptl3 is highly expressed in the hepatocytes interacting with endothelial cells.

## Discussion

DeepDoublet is an algorithm to identify two sets of single-cell transcriptome for a doublet. For this, we trained 2 FCNNs using 400,000 potential combinations of 2 single cells with various proportions. The trained FCNNs will identify two cells that comprise a doublet. With this configuration, we found neighboring cell-dependent gene expression of the hepatocytes. DeepDoublet relies on that there are enough number of cells for scRNAseq both interacting with the same cell type as well as another different cell type to train the FCNNs. Among the pool of scRNAseq, it selects the best one pair of cells or top matched pairs of cells for a doublet. Therefore, we used pcRNAseq where we have enough number of hepatocytes and LECs. It was not possible to use ProximID [10] as the number of single cells is low to train DeepDoublet.

Our approach is different from previous decomposition approaches. For instance, CIBERSORT predicted the portion of cell types from the RNA sequencing of cell population [27]. CIBERSORTx further predicted the expression levels of each cell type [28]. However, the prediction of gene expression is limited to a rough estimation of expression to a level to assign cell type. It is not an approach to interpret subtle changes in gene expression due to cell communication.

Using DeepDoublets, we showed that there are neighboring cell-dependent gene expression changes in hepatocytes. Cells show heterogeneity in gene expression. Neighboring cell-dependent gene expression may explain part of cell heterogeneity. In our test, we only found neighboring cell-dependent gene expression in hepatocytes not in LECs. LECs may not change their expression levels by the proximity of hepatocytes even though they show zonational expression. As LECs are from liver, the majority of them are possibly already influenced by the interaction with hepatocytes.

Angptl3 was found among the neighboring cell-dependent genes. Interestingly, Angptl3 is important for angiogenesis [25, 26]. The expression profiles in single cells and pcRNAseq clearly showed that Angptl3 is highly expressed in pcRNAseq, and the single cells predicted to be neighboring to LECs (Fig. 2). The hepatocytes interacting with LECs that DeepDoublet found can only be the subset of the LEC interacting hepatocytes. As shown in Fig. 2b, there still are cells in the hepatocytes group that were with high expression of Angptl3. Subsequent clustering analysis can further identify the genes that are potentially working.

We also applied DeepDoublet on PIC-seq [11] dataset to separate physically interacting cells and the cells that interact through signals in the extracellular matrix. However, we did not find neighboring-cell-dependent gene expression. In supplementary Fig. 2, a large proportion of T-DC doublets showed gene expression similar to cells under transwell and mono-culture conditions. These T-DC doublets will be decomposed into cells that do not physically interact, while we assume the cells predicted by DeepDoublet interact physically. This led to the failure when we apply DeepDoublet on the PIC-seq dataset. Moreover, PIC-seq reported some genes which are highly expressed in the cell clumps against expected expressions. However, the differences were not profound in many cases (supplementary Fig. 2).

DeepDoublet is designed for two interacting cells. More than two cells can be designed by assigning another FCNN. As there are not enough training data for more than three cells, we restricted our scope to two cells. Also, there could be more than two cells in the pcRNAseq. The application of DeepDoublet is to identify neighboring-cell-dependent gene expression, which can be done even though more than one LECs interact with hepatocytes.

Due to the characteristics of deep learning, DeepDoublet requires a substantial number of doublets for effective training. There are an increasing number of RNA sequencing datasets from physically interacting cells [29], and the size of the dataset is expected to increase. In this context, DeepDoublet can be useful by providing a tool to dissect gene expression from cell clumps.

## Supplementary Information

Additional file 1: Figure S1. The UMI count distribution for hepatocyte and liver endothelial cells. The number of UMI of the LECs is much smaller than that of the hepatocytes. Figure S2. The heatmap of the upregulated genes related to T-helper differentiation identified PIC-seq [1]. The figure showed the expression of these genes in T cells, dendritic cells (DCs), and T-DC doublets under co-culture, transwell, and mono-culture conditions for 20 h of culture. Among the genes claimed in the PIC-seq article, Foxp3 and II2 were not upregulated in any co-cultured T cell. The other genes didn't show clear differential expression in the co-cultured T cells either. Table S1. Genes upregulated in the hepatocytes that were selected by DeepDoublet. DeepDoublet predicts that these hepatocytes will interact with liver endothelial cells (LECs). Table S2. Genes downregulated in the hepatocytes that were selected by DeepDoublet. DeepDoubelt predicts that these hepatocytes will interact with liver endothelial cells (LECs).

## Data Availability

No datasets were generated or analysed during the current study. The source code is available at "*https://github.com/linbuliao/DeepDoublet*".
