# Supplementary material for: DeepDoublet identifies neighboring cell-dependent gene expression
Source: Genomics Inform. 2024 Dec 18;22:30. doi: 10.1186/s44342-024-00031-2 (PMC11654366; doi:10.1186/s44342-024-00031-2)
Supplement: Supplementary file 1 — Additional file 1: Figure S1. The UMI count distribution for hepatocyte and liver endothelial cells. The number of UMI of the LECs is much smaller than that of the hepatocytes. Figure S2. The heatmap of the upregulated genes related to T-helper differentiation identified PIC-seq [1]. The figure showed the expression of these genes in T cells, dendritic cells (DCs), and T-DC doublets under co-culture, transwell, and mono-culture conditions for 20 h of culture. Among the genes claimed in the PIC-seq article, Foxp3 and II2 were not upregulated in any co-cultured T cell. The other genes didn't show clear differential expression in the co-cultured T cells either. Table S1. Genes upregulated in the hepatocytes that were selected by DeepDoublet. DeepDoublet predicts that these hepatocytes will interact with liver endothelial cells (LECs). Table S2. Genes downregulated in the hepatocytes that were selected by DeepDoublet. DeepDoubelt predicts that these hepatocytes will interact with liver endothelial cells (LECs). [file 44342_2024_31_MOESM1_ESM.docx]

Supplementary Figures and Tables

**Table S1. Genes upregulated in the hepatocytes that were selected by DeepDoublet.** DeepDoublet predicts that these hepatocytes will interact with liver endothelial cells (LECs).

| Gene | pvals_adjusted | logFC |
| --- | --- | --- |
| Mug1 | 6.92E-12 | 0.966 |
| Angptl3 | 2.81E-15 | 0.922 |
| B2m | 5.70E-14 | 0.906 |
| Slc27a2 | 3.96E-08 | 0.904 |
| Cyp3a11 | 3.59E-12 | 0.892 |
| Pzp | 5.02E-04 | 0.846 |
| Pon1 | 5.31E-10 | 0.801 |
| Cp | 3.76E-08 | 0.791 |
| Ugt2b1 | 1.19E-09 | 0.787 |
| C4bp | 1.43E-08 | 0.782 |
| Apob | 5.20E-06 | 0.736 |
| Cpb2 | 1.50E-06 | 0.679 |
| Mup3 | 5.07E-06 | 0.677 |
| Sord | 5.75E-03 | 0.668 |
| Amy1 | 1.63E-06 | 0.656 |
| Cps1 | 1.31E-02 | 0.651 |
| Cyp2c50 | 4.97E-03 | 0.639 |
| Sephs2 | 4.99E-02 | 0.589 |
| Ugt2b34 | 2.38E-04 | 0.569 |
| Cyp2c54 | 1.29E-02 | 0.561 |
| Serinc1 | 5.45E-04 | 0.554 |
| Cyp2c67 | 1.51E-02 | 0.548 |
| Slc16a1 | 2.00E-03 | 0.52 |
| Slc17a2 | 8.71E-03 | 0.514 |
| Crot | 2.81E-02 | 0.508 |

**Table S2. Genes downregulated in the hepatocytes that were selected by DeepDoublet.** DeepDoubelt predicts that these hepatocytes will interact with liver endothelial cells (LECs).

| Gene | pvals_adjusted | logFC |
| --- | --- | --- |
| Apoc4 | 4.31E-13 | -0.953 |
| Apoc3 | 3.59E-12 | -0.867 |
| Apoc1 | 2.08E-10 | -0.771 |
| Ftl1 | 2.12E-09 | -0.753 |
| Fxyd1 | 1.28E-14 | -0.749 |
| Apoa2 | 2.08E-10 | -0.708 |
| Serpina1c | 1.78E-09 | -0.689 |
| Ttr | 1.74E-08 | -0.662 |
| Hmgcs2 | 3.76E-08 | -0.662 |
| Ass1 | 3.03E-10 | -0.64 |
| 1100001G20Rik | 2.56E-09 | -0.621 |
| Etfb | 6.78E-09 | -0.612 |
| Atp5b | 7.88E-09 | -0.612 |
| Gnmt | 2.20E-07 | -0.605 |
| Pcx | 4.05E-10 | -0.591 |
| Uqcrq | 2.19E-10 | -0.581 |
| Ndufa7 | 1.58E-08 | -0.561 |
| Ndufb7 | 3.02E-11 | -0.554 |
| Cyp2a12 | 4.05E-10 | -0.55 |
| Asl | 2.48E-09 | -0.55 |
| Atp5h | 2.08E-10 | -0.548 |
| Hamp | 2.63E-11 | -0.548 |
| Hspa8 | 6.83E-08 | -0.547 |
| Stard10 | 2.75E-10 | -0.547 |
| Ddt | 2.78E-07 | -0.539 |
| Orm1 | 8.59E-10 | -0.536 |
| Aldh1l1 | 1.78E-09 | -0.52 |
| Ttc36 | 1.32E-07 | -0.517 |
| Chchd2 | 1.27E-06 | -0.516 |
| Uqcr10 | 8.59E-10 | -0.509 |
| Cox8a | 2.82E-08 | -0.505 |
| Rpl37a | 6.23E-09 | -0.504 |

**Figure S1. The UMI count distribution for hepatocyte and liver endothelial cells.** The number of UMI of the LECs is much smaller than that of the hepatocytes.

**
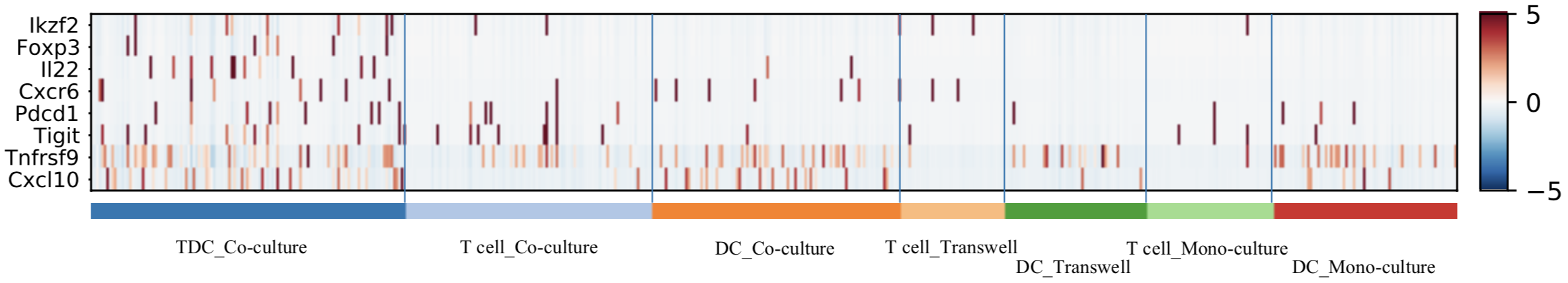
**

**Figure S2. The heatmap of the upregulated genes related to T-helper differentiation identified PIC-seq [1].** The figure showed the expression of these genes in T cells, dendritic cells (DCs), and T-DC doublets under co-culture, transwell, and mono-culture conditions for 20 hours of culture. Among the genes claimed in the PIC-seq article, Foxp3 and II2 were not upregulated in any co-cultured T cell. The other genes didn't show clear differential expression in the co-cultured T cells either.

References

1. Giladi, A.; Cohen, M.; Medaglia, C.; Baran, Y.; Li, B.; Zada, M.; Bost, P.; Blecher-Gonen, R.; Salame, T.-M.; Mayer, J. U.; David, E.; Ronchese, F.; Tanay, A.; Amit, I., Dissecting cellular crosstalk by sequencing physically interacting cells. *Nature Biotechnology* **2020,** 38, (5), 629-637.
